# Comparison of Benzene & Toluene removal from synthetic polluted air with use of Nano photocatalyticTiO_2_/ ZNO process

**DOI:** 10.1186/2052-336X-12-45

**Published:** 2014-02-05

**Authors:** Mitra Gholami, Hamid Reza Nassehinia, Ahmad Jonidi-Jafari, Simin Nasseri, Ali Esrafili

**Affiliations:** 1 Department of Environmental Health Engineering, School of Public Health, Iran University of Medical Sciences, Tehran, Iran; 2 Center for Water Quality Research (CWQR), Institute for Environmental Research (IER), Tehran University of Medical Sciences, Tehran, Iran; 3 Department of Environmental Health Engineering, School of Public Health, Tehran University of Medical Sciences, Tehran, Iran; 4 Department of Environmental Health Engineering, School of Public Health and Center for Water Quality Research (CWQR), Institute for Environmental Research (IER), Tehran University of Medical Sciences, Tehran, Iran

**Keywords:** Benzene, Toluene, TiO_2_/ZNO Nano photocatalyst, UV radiation

## Abstract

**Background:**

Mono aromatic hydrocarbons (BTEX) are a group of hazardous pollutants which originate from sources such as refineries, gas, and oil extraction fields, petrochemicals and paint and glue industries.

Conventional methods, including incineration, condensation, adsorption and absorption have been used for removal of VOCs. None of these methods is economical for removal of pollutants of polluted air with low to moderate concentrations. The heterogeneous photocatalytic processes involve the chemical reactions to convert pollutant to carbon dioxide and water. The aim of this paper is a comparison of Benzene & Toluene removal from synthetic polluted air using a Nano photocatalytic TiO_2_/ ZNO process.

**Results:**

The X-ray diffraction (XRD) patterns showed that Nano crystals of TiO_2_ and ZNO were in anatase and rutile phases. Toluene & benzene were decomposed by TiO_2_/ ZNO Nano photocatalyst and UV radiation. Kruskal-wallis Test demonstrated that there are significant differences (p_value_ < 0.05) between pollutant concentrations in different operational conditions.

**Conclusions:**

Degradation of toluene & benzene increases with increasing UV intensity and decreasing initial concentrations. Effect of TiO_2_/ZNO Nano photocatalyst on benzene is less than that on toluene. In this research, Toluene & benzene removal by TiO_2_/ZNO and UV followed first-order reactions.

## Background

Volatile organic carbon compounds create important effects in the atmosphere. They can threat human and environment, health and product photochemical oxidants and also, they contribute to stratospheric ozone depletion and the greenhouse effect [1,2].

Mono aromatics hydrocarbons (BTEX) are a group of hazardous pollutants which originate from sources such as refineries, gas, and oil extraction fields, petrochemicals and paint and glue industries [3].

Painting and insects repellent solvents based on organic compounds, especially benzene compounds, are widely used in recent years, therefore the gaseous pollutants can be released into environment [4].

Toluene is used as an octane booster agent in gasoline fuels for internal combustion engines and also as solvent, paint thinners and many chemical reactants. It is used in printing inks, adhesives, leather tanners, rubber, and disinfectants [4,5].

Toluene is found in ground and surface water, soil and air. Because of its usage in home consumptions, toluene concentration in indoor air may exceed of outdoor air. Because of exposure with both indoor levels and outdoor levels released from vehicle exhaust or unburned gasoline vapors, an average absorbed dose from inhalation exposure is estimated at about300 *μ*g/day [4].

Toluene and benzene indirectly influence climate changes as it is combined with nitrogen oxides in the presence of sunlight. Toluene also forms aerosol particles that scatter or absorb radiation and influences the formation of fog and clouds [6].

Exposure to volatile organic compounds, especially benzene compounds, causes carcinogen and acute and chronic skin diseases and even adverse effects on the nervous system. Therefore, high concentrations of them in the indoor and outdoor air has created interest in usable methods to reduce the risk involved and has become a major issue in some countries [4].

Conventional methods, including incineration, condensation, adsorption and absorption have been used for removal of VOCs. Incineration and condensation are usable only for moderate to high concentrations. Adsorption and absorption do not damage pollutants, but transfer them to another medium. None of these methods is economical for removal of pollutants of gas streams with low to moderate concentrations. Photocatalytic methods haven^,^ t the above problems and are proper for removal of pollutants with low concentrations. The heterogeneous photocatalytic processes used in pollutant degradation involve the chemical reactions to convert pollutant to carbon dioxide and water. The photocatalyst process needs to be: (a) photo-active; (b) use of ultra violet radiation (c) chemically inert (d) photo-stable and (e) inexpensive [7]. Many studies have been performed on the photocatalytic degradation of environmental pollutants by using of semiconductors, such as TiO_2_, Fe/TiO_2_, and ZNO/TiO_2_[8-13].

TiO_2_ belongs to the family of metal oxides. There are four commonly known polymorphs of TiO_2_ found in nature: anatase (tetragonal), brookite (orthorhombic), rutile (tetragonal), and TiO_2_ (B) (monoclinic) [14]. The anatase form has been found to have the most favorable characteristics for Photocatalytic oxidation(PCO), as it appears to be the most active and easiest to produce other forms. Irradiation with wavelength of 385 nm or less will generate electron–hole pairs in anatase. The anatase form is predominantly used in most commercial PCO processes [15].

### PCO of gas-phase organic compounds

Approximately 60 organic compounds have been studied in heterogeneous gas-phase PCO. Toluene (C6H5CH3) and benzene are organic compounds that have been studied in heterogeneous gas-phase PCO [16].

### Reactions mechanism

Numerous individual reactions are involved in three steps in photocatalytic processes: initiation, propagation, and termination [17]. Though photocatalytic reactions can occur in both gas and liquid phases, the focus of this paper is on those reactions where gas-phase species are reacted on solid surfaces, referred to as heterogeneous (gas/solid) photo catalysis. TiO_2_ is widely used for the degradation of a wide range of organic pollutants [18]. Many studies have shown that TiO_2_ is much more effective as a photocatalyst in the form of Nano particles than bulk powder [14]. ZNO as a potential photocatalyst has been also widely researched in recent years [11]. TiO_2_ has a large band gap, (EBG) 3.0–3.2 eV. Therefore, its activation is limited by radiation wavelengths equal to or below UV light [19]. On the other hand, ZNO is a semiconductor material with a wideband gap of 3.37 eV and high excitation energy of 60 meV at room temperature [20]. In addition, the cost of ZNO is very low and its photocatalytic efficiency is higher thanTiO_2_ for the degradation of several organic pollutants [18]. However,TiO_2_ is more environmental-resistant than ZNO [20]. Therefore, application of TiO_2_/ ZNO binary catalyst can be the way of combining the advantages of both materials and can increase the efficiency of pollutants removal. Therefore aim of this paper is comparison of removal of toluene & benzene with use of Nano photocatalytic activity of TiO_2_/ ZNO and UV radiation from synthetic polluted air.

## Materials and methods

### Experimental catalyst preparation

TiO_2_ nano photocatalyst was obtained from Degussa Co. Its specific surface area was 50 m^2^/g with a purity of 99.5%. ZNO was prepared from Nano Pars Spadana Co. Its specific surface area was 40–150 m^2^/g with a purity of 99.8%. TiO_2_ and ZNO powders were mixed (50% TiO_2_ & 50% ZNO) and dissolved in ethanol as a solvent. Then, the solution was agitated in the ultrasonic apparatus at 200 W and 59 KHz for 30 minute. The spray-coating method was used for fixation of preparedTiO_2_/ ZNO solution on the inner part of cylinder glasses with dimensions of 100*130 cm. After settlement of catalyst, the glasses were dried in the air and it was formed solid TiO_2_/ZNO layer on them. Then solid layer was fixed in furnace at 500°C for 30 minutes on glasses. Glasses were put in the reactor.

### Photocatalytic reactor set up

**Figure 1:**
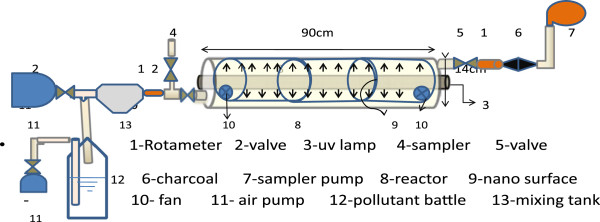
Diagram of photo reactor (falling film model).

The photocatalytic degradation of toluene was investigated in the photo reactor of TiO_2_/ZNO catalyst. Length and diameter of photo reactor were 90 and 14 cm. UV lamps with a wavelength range of 365–400 nm was employed as a UV light source in center of reactor. The experiments were conducted at pollutant different concentrations between100-200 *μ*g/m^3^ in polluted air. The reactor was designed in falling film model [21]. Inlet flow rates to photo reactor were between 0.42 -2.54 L/min. Pollutants were injected in to the reactor and then the UV lamp was turned on at different times between 5–30 minutes. Each concentration of the pollutant was exposed to UV radiation at different durations of between 5–30 minutes. Figure 1 shows photo reactor that was used.

### Analysis method

The prepared catalyst crystalline properties (TiO_2_/ ZNO) was detected by an X-ray diffraction meter (XRD). For the determination of pollutant concentration in waste air, gas samples were taken from the inlet (sampling1) and outlet (sampling2) of the photo reactor using the charcoal adsorbent tube. Gas samples with a flow rate of 0.2 L/min were acquired by sampling pump passing through the adsorbent tube at 25 min. Pollutants desorption by methanol had trace difference with CS_2_. Therefore, desorption of the toluene& benzene samples was done using methanol and then their concentration was analyzed using a gas chromatography (GC) model CP 9001, CHROM PACK company._._ The GC was equipped with FID capillary column, a flame ionization detector and Software CP Chem Stations was operated at injection temperature of 270°C, detector temperature of 250°C and oven temperature of 40°C. The GC column was 30 m capillary glass column with an inside diameter of 0.32 mm and film thickness of 0.25 mm. Helium was used as the carrier gas at a flow rate of 1.5 mL/min. The removal efficiency of the photocatalytic system was calculated by the following equation:

(1) $$\mathrm{Eff}=\frac{\mathrm{Cin}-\mathrm{Cout}}{\mathrm{Cin}}*100$$

Where the *in C* and *out C* (*μ*g/m^3^) are the toluene concentration observed at the inlet and outlet of photo reactor, respectively.

## Results

**Figure 2:**
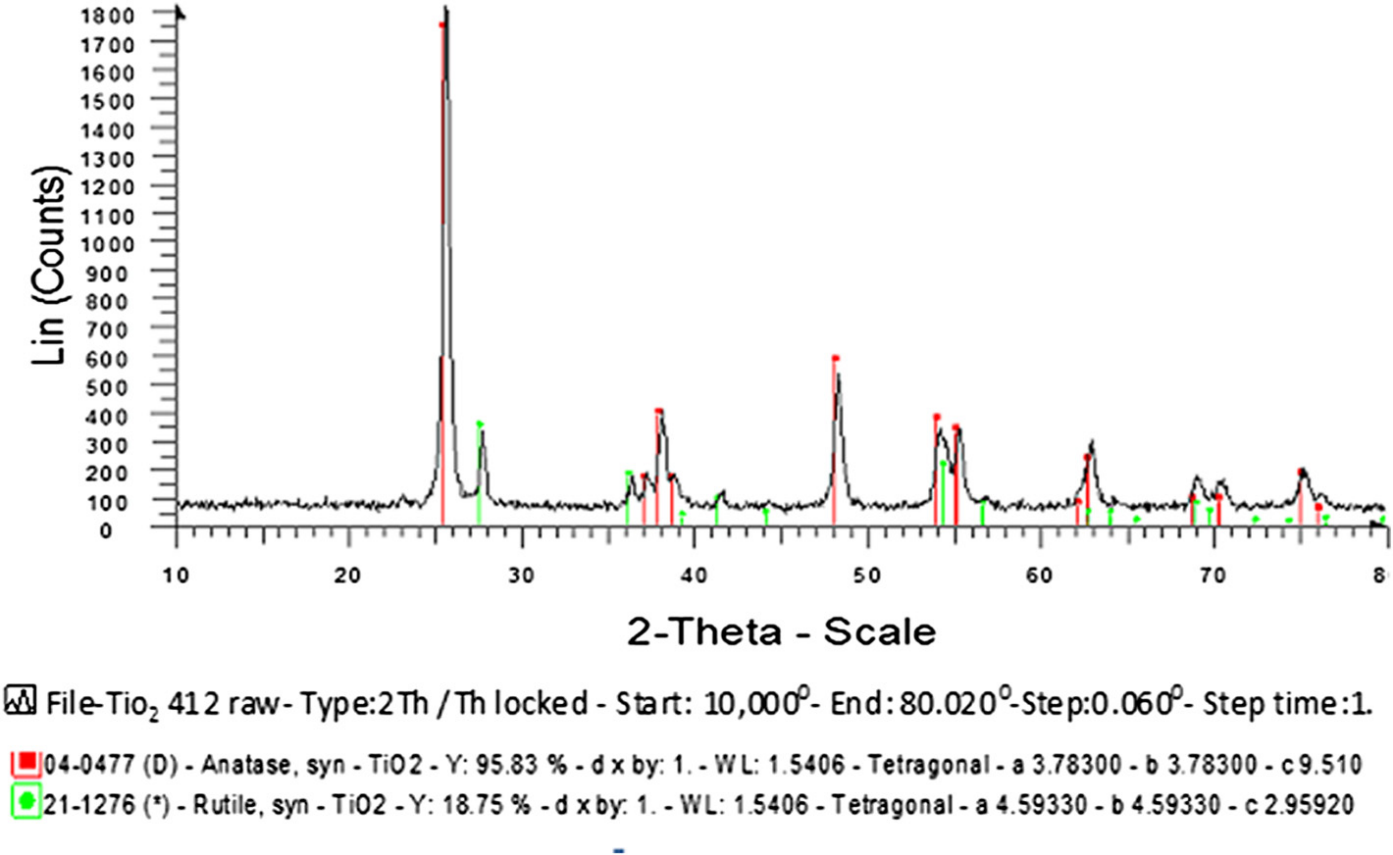
**X ray diffraction (XRD) patterns of the tio**_**2 **_**catalyst sample.**

**Figure 3:**
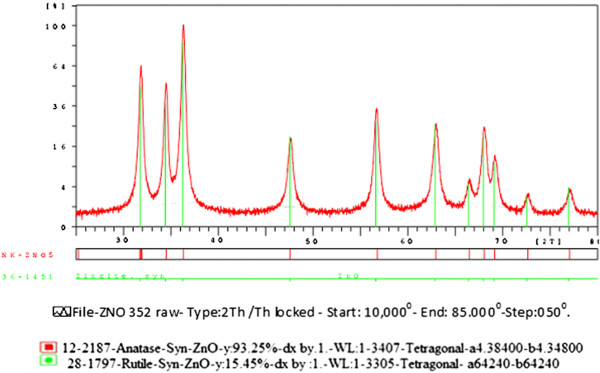
XRD pattern of ZNO catalyst nano powders.

Figures 2 and 3 show the X ray diffraction (XRD) patterns of theTiO_2_ and ZNO catalyst samples. Peak of 25° in TiO_2_ graph and peak of 32, 34, 36° in ZNO graph are maximum. Figures show that crystalline forms of TiO_2_ and ZNO were in anatase and rutile forms.

### Effect of radiation without photocatalyst

**Figure 4:**
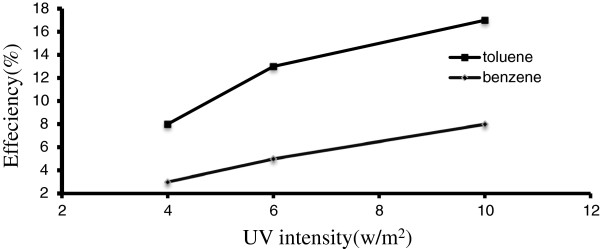
**Removal efficiency of toluene in different UV light intensities without TiO**_**2**_**/ZnO.**

Experiments were conducted at different light intensities of UV lamp light sources. UV lamp intensities were 4,6 and 10 w/m^2^. Efficient illumination of the catalyst is a critical design feature within a plug flow photocatalytic reactor. The ideally catalyst layer should be transparent to UV light to allow activation of TiO_2_/ZNO and degradation of contaminant substances. Figure 4 is a plot of removal efficiency of toluene and benzene in different UV light intensities without the use of Nano TiO_2_/ ZNO. The figure reveals that the toluene and benzene degradation is very low with radiation of UV without Nano catalyst.

### Effect of different intensities of ultra violet with photocatalyst

**Figure 5:**
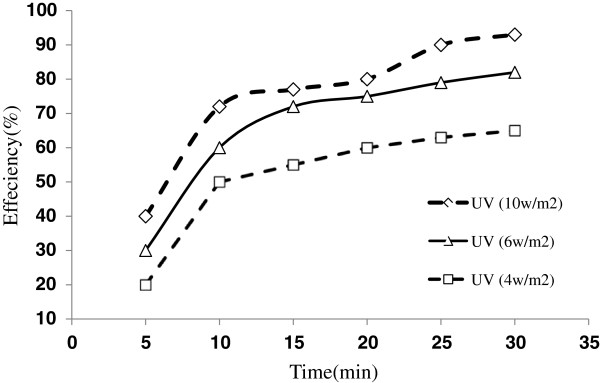
**Removal efficiency of toluene (100 μg/m**^**3**^**) with TiO**_**2**_**/ZNO photocatalyst.**

**Figure 6:**
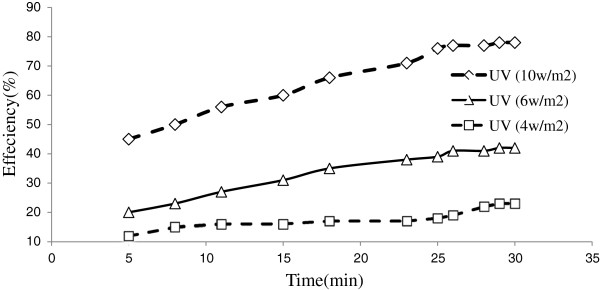
**Removal efficiency of benzene (100 μg/m**^**3**^**) with TiO**_**2**_**/ZNO photocatalyst.**

The experimental studies were done with different intensities of UV with photocatalyst in the reactor. Figures 5 and 6 represent experimental data on the removal efficiency of toluene& benzene in different UV light intensities with TiO_2_/ZNO. The figures reveal that the toluene and benzene degradation increases with increased UV lamp intensity in different times and the use of catalyst. UV lamp installed in the photo reactor with an intensity of 10 w/m^2^ provided the highest removal efficiency of toluene & benzene with TiO_2_/ZNO in the waste air; however, the removal efficiency of benzene was less than that of toluene.

### Effect of initial pollutants concentrations

**Figure 7:**
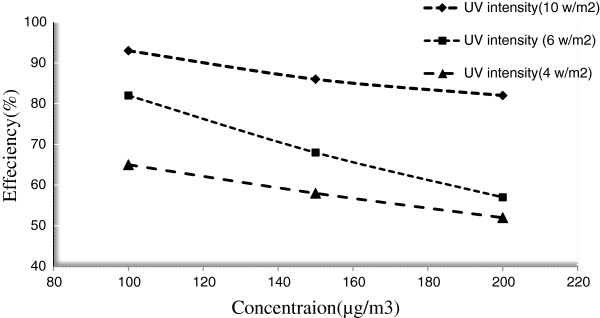
**Removal effeciency of toluene in different concentrations with TiO**_**2**_**/ZnO photocatalyst.**

**Figure 8:**
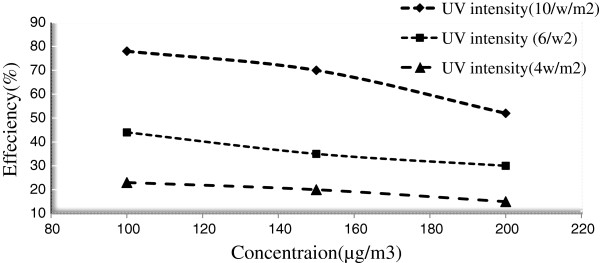
**Removal effeciency of benzene in different concentrations with TiO**_**2**_**/ZnO photocatalyst.**

Figures 7 and 8 indicate the effect of different initial concentrations of the pollutants on the removal efficiency. Experiments showed that TiO_2_/ZNO thin film excited by ultra violet radiation can remove about 93% of inlet toluene and 78% of inlet benzene in the reactor. Figures reveal that the toluene degradation decreases with increase of pollutant concentration, and that the removal percentage varies in different concentrations.

In this research, Kruskal-wallis Test demonstrated that there are significant differences (p_value_ < 0.05) between pollutant concentrations in different operational conditions.

## Discussion

Exposure to volatile organic compounds, especially benzene compounds, causes carcinogen and acute and chronic skin diseases and even adverse effects on the nervous system [4]. The heterogeneous photocatalytic processes such as the use of TiO_2_ and ZNO with UV radiation are proper for degradation of pollutants with low concentrations, especially BTEX compounds in the air [7].

Figure 4 shows that ultraviolet radiation without Nano catalyst has an insignificant effect on toluene & benzene degradation. In a research conducted by Rezaee and colleagues for the removal of toluene from air using UVc, it was reported that the removal efficiency of toluene was 6%. They represented that the reason for this removal was production of ozone and its oxidant effect [22]. In other research by Liming Yang and colleagues, paracetamol was removed up to about 12% with UV [23].

In this study, the composite TiO_2_/ZNO catalyst was successfully fixed on glass. The TiO_2_/ZNO deposited onto glass was effectively performed to get a thin film of photocatalyst. The study of toluene& benzene removal from waste air in the reactor using the prepared TiO_2_/ZNO was done to compare the effect of illumination, the contact time of the pollutant with the photocatalyst and the concentration of the pollutant. Oxidation of toluene and benzene on the photocatalyst was significantly influenced by all the parameters. From the results, it is obvious that the light intensity has a great effect on the photocatalytic reaction. This study showed that an increase of UV intensity and contemporaneous use with Nano catalyst can increase the degradation level of the pollutants. The maximum removal efficiency was observed at an ultra violet intensity of 10 w/m^2^, while the minimum level was observed at 4 w/m^2^. It has been reported that benzene can be removed by ZNO Nano photocatalyst and UV radiation [24]. TiO_2_ can absorb ultraviolet radiation of λ ≤ 387 nm (anatase) and λ ≤ 413 nm (rutile), which induces valence band (vb) electrons to the conduction band(cb) leaving positive holes (h^+^) in the valence band [25].

Possible reaction pathways have been given below:

(2) $$H_{2}0\to {\mathrm{OH}}^{-}+H^{+}$$

(3) $${H^{+}}_{\mathrm{vb}}+\mathrm{OH}\to {\mathrm{OH}}^{0}$$

(4) $$e^{-}+O_{2}\to {O_{2}}^{0}$$

(5) $${\mathrm{OH}}^{0}+\mathrm{Organic}\;\mathrm{reactants}\;+O_{2}\to \mathrm{Products}\left({\mathrm{CO}}_{2},H_{2}0,\mathrm{etc}.\right)$$

Eq 2,3,4,5 are represented as a series of reactions using TiO_2_ and ZNO as a semiconductor [7,26,27].

It has also been reported that that benzene and toluene is destructed because of the existing hydroxyl radicals [27]. It has also reported that gas-phase toluene is degraded with UV radiation and TiO_2_ catalyst [5].

In another study, TiO_2_-SiO_2_ based photocatalysts were used for the removal of toluene and it was demonstrated that the porous photocatalyst with very high adsorption capacity enhanced the subsequent photocatalysis reactions and lead to appositive synergistic effect [7]. Another study of benzene removal from the air concluded that benzene is removed by the influence of ultraviolet at a wavelength of 365 nm and ZNO photocatalyst [10]. In another study, the dependency of the toluene removal efficiency of several key influence factors (UV light intensity, and photocatalyst loading) was studied in the photocatalytic reactor. The results showed that all parameters play an important role in the oxidation of toluene, and that the catalyst could be regenerated by UV irradiation.

Results showed that the removal efficiency of benzene is less than toluene. It has been shown that benzene is less susceptible to photocatalytic oxidation than toluene [17]. It has been reported that toluene is 13 times more reactive than benzene with chlorine radicals, but much closer reactivity between toluene and benzene was observed with only hydroxyl radicals present [16].

**Figure 9:**
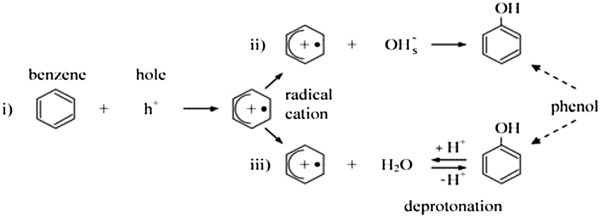
Route of benzene degradation by direct hole oxidation.

**Figure 10:**
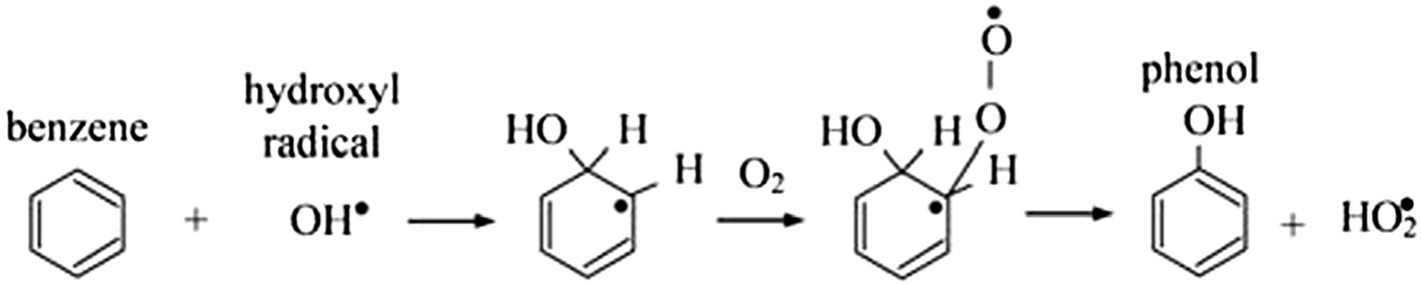
**Route of benzene degradation by OH**^**0 **^**radical addition.**

The difference could partially explain why the presence of chlorine does not enhance the degradation rate of benzene. Two routes have been proposed for benzene degradation: 1. (a) Direct hole oxidation followed by reaction of the resulting radical cation either(b) with a surface basic OH group, or(c) with an adsorbed water molecule and subsequent deprotonation to yield phenol, the major intermediate detected. 2. OH^0^ radical addition to yield a cyclohexa dienyl radical [7,11,16]. Figures 9 and 10 show routes of benzene degradation

The kinetic of photocatalytic oxidation processes has usually been discussed according to the following Langmuir –Hinshelwood kinetic model [7].

(6) $$\mathrm{rR}=\frac{-\mathrm{dCR}}{\mathrm{dt}}=\frac{\mathrm{KrKCR}}{1+\mathrm{KCR}}$$

r_R:_ reaction velocity (mg/L.min) C_R_: concentration(mg/L)

k_r_: constant of velocity(mg/L/min) k: absorption coefficient (L/mg)

In the case of low initial concentration of pollutant, the L–H kinetic equation could be noted to be a follow first order rate equation [7].

(7) $$\frac{\mathrm{dC}}{\mathrm{dT}}=-\mathrm{KT}$$

or

(8) $$\ln\left(\frac{C}{\mathrm{Co}}\right)=-\mathrm{kt}$$

**Figure 11:**
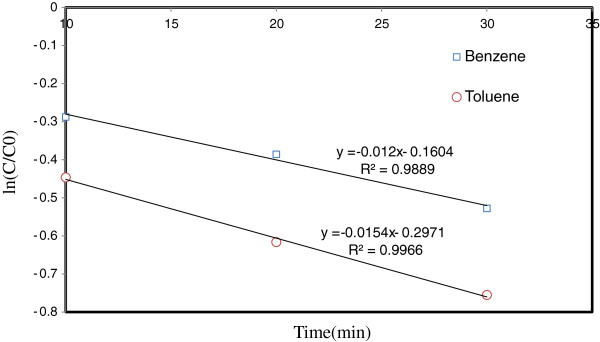
**Kinetic reactions of toluene & benzene removal by TiO**_**2**_**/ZNO and UV based on first order reactions.**

Figure 11 shows kinetic reactions of toluene & benzene emoval by TiO_2_/ZNO and UV based on first-order reactions. As can be seen in the figure, the quantity of R^2^ calculated in first-order reaction graph is the maximum; therefore, in this research, toluene& benzene removal by TiO_2_/ZNO and UV follows first-order reactions, which was also reported by previous studies [28].

## Conclusions

Degradation of toluene & benzene increases with increasing UV intensity and decreasing initial concentrations. TiO_2_/ZNO thin film increased the photocatalytic activity due to the maximization of UV lamp illumination and increasing of retention time of toluene & benzene in the reactor. However, the effect of TiO_2_/ZNO Nano photocatalyst on benzene is less than that on toluene. In this research, Toluene & benzene removal by TiO_2_/ZNO and UV followed first-order reactions.

## Abbreviations

BTEX: Benzene, Toluene, Ethyl benzene, Xylene.

## Competing interest

The authors declare that they have no competing interest.

## Authors’ contributions

MG, HRN, AJJ, SN, AE: Contributed equally in the literature search, figures, study design, data analysis, data interpretation, writing etc, in the final manuscript. All authors read and approved the final manuscript.
